# Does Kisspeptin Belong to the Proposed RF-Amide Peptide Family?

**DOI:** 10.3389/fendo.2014.00134

**Published:** 2014-08-13

**Authors:** Seongsik Yun, Dong-Kyu Kim, Michael Furlong, Jong-Ik Hwang, Hubert Vaudry, Jae Young Seong

**Affiliations:** ^1^Graduate School of Medicine, Korea University, Seoul, South Korea; ^2^INSERM U982, University of Rouen, Mont-Saint-Aignan, France

**Keywords:** kisspeptin, RF-amide, spexin, galanin, coevolution, gene duplication, evolutionary history

## Abstract

Kisspeptin (KISS) plays a key role in regulating reproduction by binding to its receptor, GPR54. Because of the Arg-Phe (RF) sequence at its carboxyl terminus, KISS has been proposed to be a member of the RF-amide peptide family consisting of neuropeptide FF (NPFF), neuropeptide VF (NPVF), pyroglutamylated RF-amide peptide (QRFP), and prolactin-releasing hormone (PRLH). Evolutionary relationships of protein families can be determined through phylogenetic analysis. However, phylogenetic analysis among related peptide families often fails to provide sufficient information because only short mature peptide sequences from full preprohormone sequences are conserved. Considering the concept of the coevolution of peptide ligands and their cognate receptors, evolutionary relationships among related receptor families provide clues to explore relationships between their peptides. Although receptors for NPFF, NPVF, and QRFP are phylogenetically clustered together, receptors for PRLH and KISS are on different branches of the phylogenetic tree. In particular, KISS has been proposed to be a member of the KISS/galanin/spexin family based on synteny analysis and the phylogenetic relationship between their receptors. This article discusses the evolutionary history of the receptors for the proposed RF-amide peptide family and proposes that, from an evolutionary aspect, KISS has emerged from an ancestor, which is distinct from those of the other RF-amide peptides, and so should be classed separately.

## Introduction

**Figure 1:**
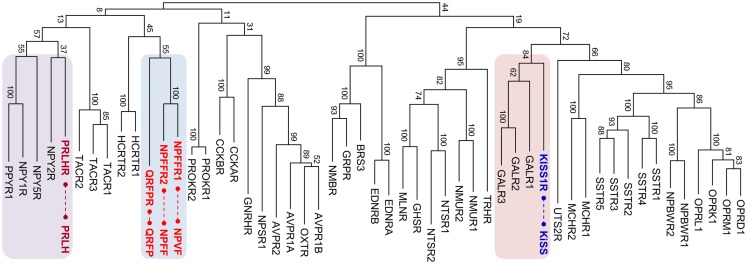
**Maximum likelihood phylogenetic tree for human rhodopsin-like neuropeptide GPCRs**. The amino acid sequences of the receptors were aligned using MUSCLE, and the tree was constructed by MEGA 5.1. The RF-amide peptide receptor groups are indicated with colored boxes and corresponding peptides are connected by dashed lines. Bootstrap values represent 100 replicates. MLNR, motilin receptor; GHSR, ghrelin receptor; NTSR, neurotensin receptor; NMUR, neuromedin U repceptor; TRHR, thyrotropin-releasing hormone receptor; NMBR, neuromedin B receptor; GRPR, gastrin-releasing peptide receptor; BRS, bombesin receptor; EDNR, endothelin receptor; PRLHR, prolactin-releasing hormone receptor; NPYR, neuropeptide Y receptor; PROKR, prokineticin receptor; TACR, tachykinin receptor; CCKR, cholecystokinin receptor; HCRTR, hypocretin (orexin) receptor; QRFPR, pyroglutamylated RFamide peptide receptor; NPFFR, neuropeptide FF receptor; AVPR, arginine vasopressin receptor; OXTR, oxytocin receptor; NPSR, neuropeptide S receptor; GnRHR, gonadotropin-releasing hormone receptor; UTSR, urotensin receptor; GALR, galanin receptor; KISSR, kisspeptin receptor; MCHR, melanin-concentrating hormone receptor; SSTR, somatostatin receptor; NPBWR, neuropeptides B/W receptor; OPR, opioid receptor.

The RF-amide peptides that harbor the Arg-Phe-amide sequence in their carboxyl (C)-termini were first discovered in a species of mollusk, *Macrocallista nimbosa* (1). Following this discovery, various RF-amide peptides have been identified in other invertebrate species (2, 3). In vertebrates, neuropeptide FF (NPFF) along with its receptor GPR74 (NPFFR2) was the first to be identified in the central nervous system of mammals (4). Fifteen years later, paralogous peptides, gonadotropin-inhibitory hormone (GnIH) in quail brain (5), and neuropeptide VF (NPVF) in mammals (6, 7) were identified. Currently, GnIH and NPVF are found to be orthologous to each other and activate the receptor GPR147 (NPFFR1) (6–10). Another paralogous peptide, pyroglutamylated RF-amide peptide (QRFP or 43RF-amide peptide), and its receptor GPR103 (QRFPR) have been identified in the hypothalamus and spinal cord of mammals (11–13). The receptors for these three vertebrate RF-amide peptides, NPFFR1, NPFFR2, and QRFPR, are phylogenetically very close to each other (Figure 1), indicating an evolutionarily common origin for these receptors. The other RF-amide-related peptides in vertebrates are prolactin-releasing hormone (PRLH or PrRP) and kisspeptin (KISS). Interestingly, the receptors for PRLH (PRLHR) and KISS (KISSR) are phylogenetically distant from NPFFR1, NPFFR2, and QRFPR. They are more closely related to neuropeptide Y (NPY) receptors (14, 15) and galanin (GAL) receptors (16), respectively (Figure 1).

The RF-amide peptides do not exhibit any sequence similarity to each other, other than the presence of the common RF-amide at their C-termini (17). As most neuropeptides have coevolved with their cognate receptors (18, 19), phylogenetic analysis of the related receptor families may mirror the evolutionary history of the peptide families (20, 21). Phylogeny of the RF-amide peptide receptor family, however, revealed distant relationships between PRLHR, KISSR, and the other RF-amide receptors (15, 16). This suggests that PRLH and KISS, at least, are likely to have originated from ancestors different from that of NPFF, NPVF, and QRFP. Therefore, it is timely to redefine the RF-amide family group members according to the evolutionary histories of individual RF-amide peptides. This article reviews the evolutionary relationships between KISS, and the other RF-amide peptides along with their paired receptors, and proposes that KISS is independent from the RF-amide peptide family.

## General Mechanism for the Evolution of Neuropeptides and Their Receptors

Neuropeptide and receptor families have expanded through whole-genome duplications (WGD) and local tandem gene duplications before and after WGD during vertebrate evolution (14, 16, 20, 22–26). To date, synteny analyses of vertebrate genome fragments and comparison of entire chromosomes of evolutionarily distinct taxa support two rounds (2R) of WGD during early vertebrate evolution. These events produced four paralogous chromosomal regions (paralogons) sharing similar sets of genes (27–29). A third round (3R) of WGD during an early phase of bony fish emergence resulted in octupled paralogons in teleosts (30).

The evolutionary history of a gene family can be traced by phylogenetic analysis. However, in the case of peptide gene families, phylogenetic analysis often does not provide sufficient information to conclude the evolutionary relationship among related peptide gene families (20, 31). In general, signal peptide sequences are not conserved and propeptide sequences, other than the mature peptide, are highly variable because they are free from evolutionary selective pressure (32). Sequence comparison of only a short, conserved mature peptide is not enough to extrapolate correct relational information. Furthermore, paralogous peptide genes that arose by local gene duplication before 2R exhibit considerable variation even in the mature peptide sequences (20, 31). For instance, the mature peptide sequences of the ligand genes for class B (secretin-like) G-protein-coupled receptors (GPCR) are highly variable (20, 31) while maintaining conserved three-dimensional structure to a general extent (33). Thus, alternative methods need to be applied to explore the evolutionary relationships among peptide gene families.

In contrast to peptide genes, evolutionary relationships among related receptor families can be readily assessed by phylogenetic analysis since the transmembrane domains of the receptors are relatively conserved across vertebrate species and the amino acid sequences of receptors are long enough to generate a convincing phylogenetic tree. Thus, tracing relationships among related receptor families can provide clues to understand the evolutionary history of their corresponding peptide genes. For instance, the evolutionary histories of receptors for secretin family peptides, including corticotropin-releasing hormone, calcitonin, parathyroid hormone, glucagon, and secretin subfamilies may allow us to speculate about possible evolutionary histories for the cognate peptide genes, for which the sequence similarities are not well preserved (20, 25, 31). In addition, locating related peptide genes on the reconstructed pre-2R ancestral chromosomes (or linkage group) is an alternative tool to explore the relationships among related gene families (20, 34). Paralogous genes that emerged through local duplications before 2R reside in the same vicinity on the pre-2R linkage group. For instance, many secretin family peptide genes are located on the same pre-2R linkage group, indicating close evolutionary relationships among the peptide genes (20). The gene families of the neuropeptides, KISS, GAL, and spexin (SPX), are in the vicinity of the same pre-2R linkage group (16). This possibility is further supported by phylogenetic relationships among the receptors of these peptides (Figure 1) (16). The neuropeptide Y (*NPY*) gene and its paralogous genes, including peptide YY (*PYY*) and pancreatic polypeptide (*PPY*), reside on the same chromosome or on paralogons (35, 36). Likewise, the NPY receptor (NPYR) family seems to have been generated by local gene duplications followed by 2R WGD (14, 15, 37).

## Evolutionary History of NPFF/NPVF, QRFP, and PRLH

Neuropeptide NPFF was the first RF-amide peptide characterized in the central nervous system of mammals (4, 8, 38, 39). NPFF is known to be involved in morphine tolerance, adipogenesis, and anorectic activity (40–45). In 2000, Tsutsui et al. found a new RF-amide peptide, GnIH, in quail brain (5, 46). This turned out to be an ortholog of NPVF in humans (9, 47). Albeit with some cross-reactivity, NPFF has a high affinity to NPFFR2 (4) while NPVF more selectively activates NPFFR1 (6, 48, 49).

The neuropeptides NPFF and NPVF seem to be 2R-generated paralogs (ohnologs) as their genes are located on two 2R-generated paralogons of human chromosomes. *NPFF* is located in the vicinity of the neuropeptide *tachykinin 3* (*TAC3*) gene on human chromosome 12 while *NPVF* is on human chromosome 7, which also contains the *TAC1* gene. Likewise, the receptors *NPFFR1* and *NPFFR2* are ohnologous to each other since *NPFFR1/TACR2* and *NPFFR2/TACR3* pairs are on paralogons of human chromosomes 10 and 4, respectively (6, 15, 50). It is of interest to note that human chromosome 10 also harbors *PRLHR* and *PPYR* (a receptor for NPY family peptides) and that human chromosome 4 has *QRFPR* and three *NPYRs, NPY1R, NPY2R*, and *NPY5R* (14, 15, 51). The phylogenetic tree in Figure 1 also shows that these receptors are clustered together. These observations suggest that *NPFFRs, QRFPR, TACRs, NPYRs*, and *PRLHR* may have emerged through local duplications from a common ancestor before 2R and expanded their members via 2R. Although the hypocretin (orexin) receptor (HCRTR) is shown in that branch, the *HCRTR* gene is not shown in the paralogons. This may be due to translocation of the gene to other chromosomal regions during evolution (20). Similarly, many peptide genes for these receptors are clustered on 2R-generated paralogons. For instance, the *HCRT, PYY*, *PPY*, and *TAC4* genes are closely located on a region of human chromosome 17, which is likely another paralogon of *NPFF/TAC*-containing regions of human chromosomes 7 and 12 (15). Thus, it seems likely that these peptide genes arose during vertebrate evolution in a manner similar to that of receptor genes. It is noteworthy that the chicken C-RF-amide peptide (*PRLH2*), an ohnolog of *PRLH* resides near *NPY*, *TAC1*, and *NPVF* on the chromosomes of chickens, medaka, and tetraodon (52, 53). These findings suggest the presence of evolutionary relationships between the NPFF family and the other RF-amide peptides QRFP and PRLH, along with non-RF-amide peptides such as NPY, TAC, and HCRT.

Pyroglutamylated RF-amide peptide (43RF-amide), a long form of 26RF-amide, has been discovered in the hypothalamus and spinal cord of humans (11–13, 54). Intravenous or intracerebroventricular administration of QRFP increased plasma aldosterone levels and food intake in rats (11, 12, 55). While humans have only one QRFP receptor (12, 13), rodents have two receptors QRFPR1 and QRFPR2 (56, 57). QRFPR2 appears to be an ohnolog of QRFPR1. A region of mouse chromosome 6 has *QRFPR2* and *TACR1*, indicating that this region is another paralogon for the *QRFPR/NPFFR/TACR*-containing chromosomal regions as described previously. As the phylogenetic tree reveals a close relationship between QRFPR and NPFFRs, an evolutionarily close relationship between QRFR and NPFF/NPVF can be postulated.

Prolactin-releasing hormone was first identified by a reverse pharmacological approach in 1998 (58). Physiological roles of PRLH include regulation of stress response (59–61), reduced appetite (62–64), and stimulation of luteinizing hormone and follicle stimulating hormone (65). The orphan receptor GPR10 was identified as the receptor for PRLH (58, 66, 67). PRLHR exhibits a close relationship in its sequence identity with the NPYR family (14, 15). *NPY4R*, a member of the NPYR family is positioned together with *PRLHR1* on the same chromosome of humans and chickens (15). There are also some structural similarities between PRLH and NPY family peptides. Both *PRLH* and *NPY* family genes have two coding exons. The first coding exon contains a signal peptide sequence followed by the N-terminal region of the mature peptide sequence. The second coding exon contains the C-terminal region of the mature peptide with conserved Arg-Phe-Gly (RFG) or Arg-Tyr-Gly (RYG) residues followed by a dibasic cleavage site. Like PRLH, some NPY peptide family members such as bovine PYY and PPY have RF-amide sequences, while NPY, PPY, and PYY from most vertebrates contain the Arg-Tyr-amide (RY-amide) sequence (15, 68, 69). It is also interesting to note that the NPY peptide is able to activate PRLHR1 at micromolar levels (15). These observations together with phylogenetic analysis of neuropeptide receptors suggest that the *PRLH/NPY* family genes and their receptor genes emerged by local gene duplications during early vertebrate evolution (14, 15). These local duplication events are likely to have occurred after the genes split from their common ancestors into the PRLH/NPY and NPFF/QRFP systems.

## Coevolution of the Spexin/Galanin/Kisspeptin Family

The *KISS* gene was first identified as a tumor suppressor gene expressed in human melanoma and breast cancer cells (70, 71). Later, the *KISS* gene was found to produce a functional peptide with an RF-amide sequence (designated as kisspeptin or metastin) that activates an orphan GPCR, GPR54 (KISSR) (72–74). KISS and KISSR are involved in the onset of puberty and the control of reproduction and food consumption (75–80). After the discoveries of mammalian *KISS* and *KISSR*, two paralogs of the *KISS* gene (*KISS2* and *KISS3*) and three paralogs of the *KISSR* gene (*KISSR2, KISSR3*, and *KISSR4*) have been identified in a variety of vertebrate species (32, 81–83). All these paralogs seem to be ohnologs as each of them is located on 2R-generated paralogons (32, 82, 83).

**Figure 2:**
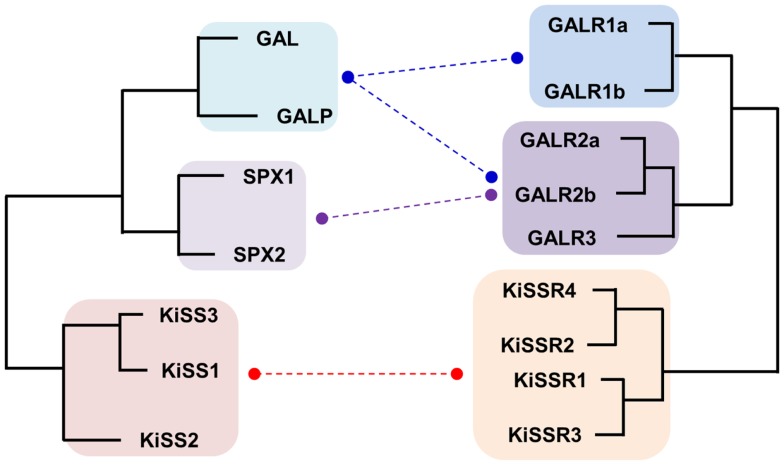
**Schematic diagram for the evolutionary relationship between KISS/GAL/SPX family peptides and their corresponding receptors in vertebrates**. Paralogs for each peptide and receptor found in vertebrates are included. Functional interaction between the ligands and receptors are indicated by dashed lines. The KISS peptides are able to activate all types of KISSRs with some differences in their ligand potencies but they do not activate any type of GALRs. GALs exhibit high potencies toward GALR1 and GALR2 family receptors but low potencies toward GALR3. SPXs activate GALR2 and GALR3 with high affinities but do not activate GALR1.

Although KISS has been acknowledged as a member of the RF-amide peptide family, no supporting evidence, other than the presence of the RF-amide sequence in its C-terminus, has been provided. KISSR was originally reported to have considerably high degree of sequence similarity with galanin receptors (GALR) (84). This result raises the possibility that KISS/KISSR and GAL/GALR pairs have diverged from a common ancestor and not from the RF-amide peptide/receptor ancestors. Recently, it was shown that the novel neuropeptides spexin1 (SPX1) and spexin2 (SPX2) are functional ligands for GALR2 and GALR3 but not GALR1 (16, 85). In particular, SPXs are more potent than GAL in activation of GALR3, while they show potencies to GALR2 similar to that of GAL (16). Synteny analysis and relocation of the gene families on the reconstructed vertebrate ancestral linkage groups show that *SPX, GAL*, and *KISS* family genes are distributed among 2R-generated 4 paralogons (4 linkage groups). Three linkage groups contain *SPX1* and *KISS2, SPX2* and *GAL*, and *KISS3* and galanin-like peptide (*GALP*), respectively. The fourth linkage group has *KISS1* alone (16, 86). This study proposed that ancestral forms of *KISS, GAL*, and *SPX* arose by tandem local duplications before 2R and expanded their family members through 2R (16). Likewise, KISSRs and GALRs are likely to have emerged through local duplications before 2R, producing three subgroups in vertebrates: KISSRs (4 KISSRs), GALR1 (GALR1a and GALR1b), and GALR2/3 (GALR2a, GALR2b, and GALR3) (Figure 2). Altogether, these results suggest that the evolutionary origin of the KISS/SPX/GAL family is far distant from those of the NPFF/QRFP and PRLH/NPY families.

## Conclusion

Rapid accumulation of genomic sequence information from various invertebrate and vertebrate species and the development of bioinformatic tools, including phylogenetic analysis, small scale genome comparison to identify orthologous and paralogous relationships of genes, and reconstruction of ancient chromosomes, have facilitated exploration of the relationships and origins of peptide and receptor gene families (20, 28, 32, 34, 87, 88). Based on the phylogenetic analysis of neuropeptide receptors and the syntenic relationships of neuropeptide genes, we suggest that the proposed RF-amid peptide family arose from three different ancestors. NPFF and NPVF are likely 2R-generated paralogs and have a close evolutionary relationship with QRFP. PRLH is evolutionarily closer to the NPY family than the NPFF/NPVF/QRFP group. KISS is likely a member of the KISS/GAL/SPX peptide family and their evolutionary origin is far distant from those of the other RF-amide peptides. This study may provide an insight into the mechanism for coevolution of neuropeptides and their receptors.

## Conflict of Interest Statement

The authors declare that the research was conducted in the absence of any commercial or financial relationships that could be construed as a potential conflict of interest.
